# Left ventricular hypertrophy and metabolic resetting in the *Notch3*-deficient adult mouse heart

**DOI:** 10.1038/s41598-023-42010-7

**Published:** 2023-09-12

**Authors:** Francesca Del Gaudio, Dongli Liu, Maarja Andaloussi Mäe, Eike-Benjamin Braune, Emil M. Hansson, Qing-Dong Wang, Christer Betsholtz, Urban Lendahl

**Affiliations:** 1 Department of Cell and Molecular Biology, Karolinska Institutet, Stockholm, Sweden; 2 Department of Immunology, Genetics, and Pathology, Rudbeck Laboratory, Uppsala University, Uppsala, Sweden; 3 Bioscience Cardiovascular, Research and Early Development, Cardiovascular, Renal and Metabolism (CVRM), BioPharmaceuticals R&D, AstraZeneca, Gothenburg, Sweden; 4 Department of Medicine, Karolinska Institutet, Huddinge, Sweden; 5 Present Address: Department of Pediatrics at the First Affiliated Hospital, Guangxi Medical University in Nanning, Guangxi, People’s Republic of China

**Keywords:** Molecular biology, Cell signalling

## Abstract

The heart depends on a functional vasculature for oxygenation and transport of nutrients, and it is of interest to learn how primary impairment of the vasculature can indirectly affect cardiac function and heart morphology. *Notch3*-deficiency causes vascular smooth muscle cell (VSMC) loss in the vasculature but the consequences for the heart remain largely elusive. Here, we demonstrate that *Notch3*^*-/-*^ mice have enlarged hearts with left ventricular hypertrophy and mild fibrosis. Cardiomyocytes were hypertrophic but not hyperproliferative, and the expression of several cardiomyocyte markers, including *Tnt2*, *Myh6*, *Myh7* and *Actn2,* was altered. Furthermore, expression of genes regulating the metabolic status of the heart was affected: both *Pdk4* and Cd36 were downregulated, indicating a metabolic switch from fatty acid oxidation to glucose consumption. *Notch3*^*-/-*^ mice furthermore showed lower liver lipid content. *Notch3* was expressed in heart VSMC and pericytes but not in cardiomyocytes, suggesting that a perturbation of Notch signalling in VSMC and pericytes indirectly impairs the cardiomyocytes. In keeping with this, *Pdgfb*^ret/ret^ mice, characterized by reduced numbers of VSMC and pericytes, showed left ventricular and cardiomyocyte hypertrophy. In conclusion, we demonstrate that reduced Notch3 or PDGFB signalling in vascular mural cells leads to cardiomyocyte dysfunction.

## Introduction

Notch signalling is an evolutionarily conserved signalling mechanism, which regulates development and homeostasis of most organs in the body, including the cardiovascular system. Activation of Notch signalling ensues when a transmembrane Notch receptor on a signal-receiving cell interacts with transmembrane Notch ligands presented on a juxtaposed signal-sending cell^1^. Ligand-receptor interaction results in proteolytic processing of the Notch receptor by cleavage of the receptor just outside the plasma membrane, followed by a final proteolytic cleavage in the plasma membrane, the latter executed by the γ-secretase complex. The final cleavage steps results in the liberation of the C-terminal portion of the Notch receptor, the Notch intracellular domain (Notch ICD), which relocates from the plasma membrane to the cell nucleus. In the nucleus, Notch ICD interacts with the DNA-binding protein CSL (a.k.a. RBPj) and MAML1, forming a ternary transcriptional complex that regulates expression of Notch downstream genes, including the genes encoding Hes and Hey transcription factors^2^.

Mutations in the Notch pathway that cause heart disease have been identified^3^. Notably, *NOTCH1* mutations are observed in bicuspid aortic valve disease^4^ and hypoplastic left heart syndrome^5^, while mutations in the *MIB1* gene, encoding an E3 ubiquitin ligase important for Notch ligand presentation, are linked to left ventricle cardiomyopathy^6^. Furthermore, most patients with Alagille syndrome (OMIM No. 118450), a genetic disorder caused by mutations in the Notch ligand Jagged1, exhibit the congenital heart disease Tetralogy of Fallot^7^. Notch signalling is also important for formation of septa and valves during the genesis and development of the heart^8^, and impaired Mib1 function causes left ventricular non-compaction myocardiopathy and bicuspid aortic valve in the mouse^9^.

In addition to its role in heart development, Notch signalling plays a pivotal role for development and homeostasis of the vasculature^10^ and the *Notch3* gene is particularly important in this regard. *Notch3* is predominantly expressed in mural cells (VSMC and pericytes)^11–13^ and *Notch3*-deficient mice exhibit a progressive loss of VSMC after birth^14,15^. *NOTCH3* missense mutations cause the most common monogenic brain small vessel disease CADASIL (Cerebral Autosomal Dominant Arteriopathy with Subcortical Infarcts and Leukoencephalopathy; OMIM No. 125310)^16^. The vast majority of *NOTCH3* CADASIL mutations are cysteine-altering^17,18^, but cysteine-sparing *NOTCH3* mutations have also been linked to brain vascular disease^19,20^, further underscoring the importance of NOTCH3 function for the brain vasculature.

While the progressive loss of VSMC in the *Notch3*^*-/-*^ mice is well established^14,15^, less is known about consequences in other organs, including the heart. Here, we report that *Notch3*^*-/-*^ mice exhibit left ventricular hypertrophy, cardiomyocyte hypertrophy and mild fibrosis. Gene expression changes indicating a metabolic switch from fatty acid oxidation to glucose consumption were observed, and the lipid content in the liver was reduced. As *Notch3* was expressed in VSMC and pericytes but not in cardiomyocytes, the data suggest that the impairment of the cardiomyocytes is indirect and that specific cellular perturbations in the vasculature are sufficient to produce a cardiomyocyte phenotype.

## Results

### Left ventricular hypertrophy in ***Notch3***^-/-^ mice

**Figure 1:**
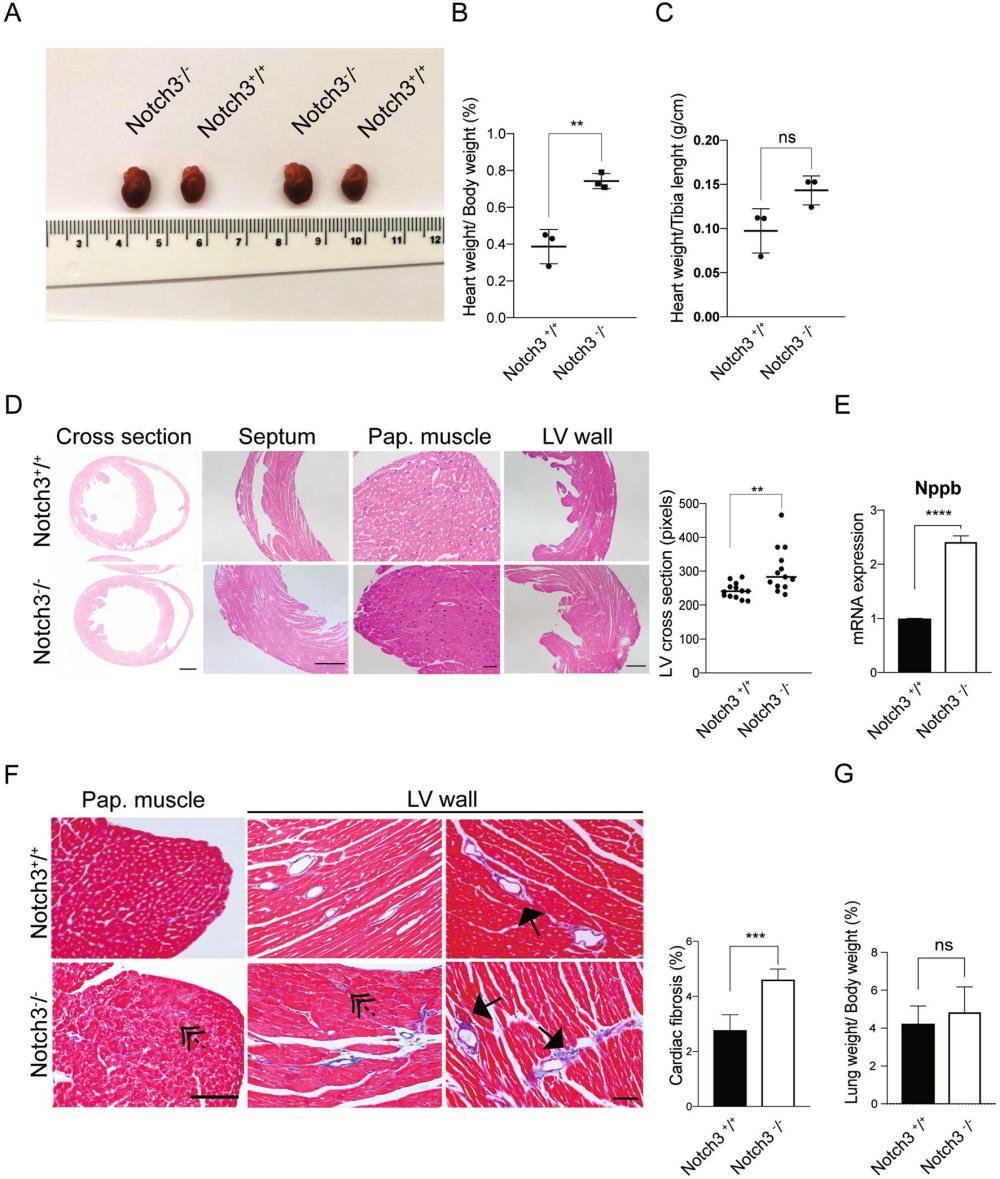
*Notch3*^*-/-*^ mice exhibit left ventricular hypertrophy. (**A**) The size of the hearts in control (*Notch3*^+*/*+^) and *Notch3*^*-/-*^ mice. (**B**) Heart/body weight ratio (%) in control and *Notch3*^*-/-*^ mice. (**C**) Heart weight/tibia length ratio (g/cm) in control and *Notch3*^*-/-*^ mice. (**D**) Sections from septum, papillary muscles and left ventricular wall (LV wall) from control and *Notch3*^*-/-*^ mice. Size bars: cross section, 200μm; septum and papillary muscle = 200 μm; LV wall = 50 μm. Quantification of the length of the cross section of the left ventricular wall. N = 3 animals per genotype were analysed and a minimum of 4 fields of image per mouse were used for quantification. (**E**) *Nppb* mRNA expression from n = 3 control and n = 3 *Notch3*^*-/-*^ hearts. (**F**) Analysis of fibrosis in cross-sections of left ventricle from control and *Notch3*^*-/-*^ mice; solid arrows indicate perivascular fibrosis and dotted arrows denote interstitial fibrosis. Size bar = 20 μm. Quantification of cardiac fibrosis is presented to the right. N = 4 animals per genotype were used. (**G**) Analysis of the weight of lungs from n = 6 control and n = 6 *Notch3*^*-/-*^ mice. The mice used for these experiments were 10–14 months old. **p < 0.01, ***p < 0.001, ****p < 0.0001, ns = non-significant.

To learn whether the heart was affected by loss of Notch3 function, we measured the weight of the hearts in *Notch3*^*-/-*^ mice. *Notch3*^*-/-*^ hearts were enlarged (Fig. 1A), with an increased heart versus body weight ratio (Fig. 1B) and a trend towards increased heart weight versus tibia length ratio (Fig. 1C). These data corroborate a previous report of cardiac hypertrophy in a different *Notch*3 knockout mouse model^21^.

Analysis of the morphology of *Notch3*^*-/-*^ and control hearts demonstrated that the left ventricle and papillary muscles from the *Notch3*^*-/-*^ mice were thickened, whereas the size of the septum was not significantly altered (Fig. 1D). Expression of the gene *Nppb*, which encodes the brain natriuretic peptide (BNP) and is upregulated when the ventricles are subjected to increased filling pressures and left ventricle wall stretch^22^, was increased in the *Notch3*^*-/-*^ hearts (Fig. 1E), corroborating the notion of left ventricular hypertrophy. Compared to wildtype control mice, mild fibrosis and perivascular and interstitial collagen deposition were observed in the *Notch3*^*-/-*^ hearts (Fig. 1F). In contrast, the weight of *Notch3*^*-/-*^ lungs did not differ from control mice (Fig. 1G) nor exhibited any signs of fibrosis or vascular changes (data not shown). Collectively, these observations suggest that *Notch3*^*-/-*^ mice exhibit left ventricular hypertrophy, combined with mild fibrosis.

### Left ventricular hypertrophy in *Notch3*^-/-^ mice is associated with cardiomyocyte hypertrophy but not cardiomyocyte hyperproliferation

**Figure 2:**
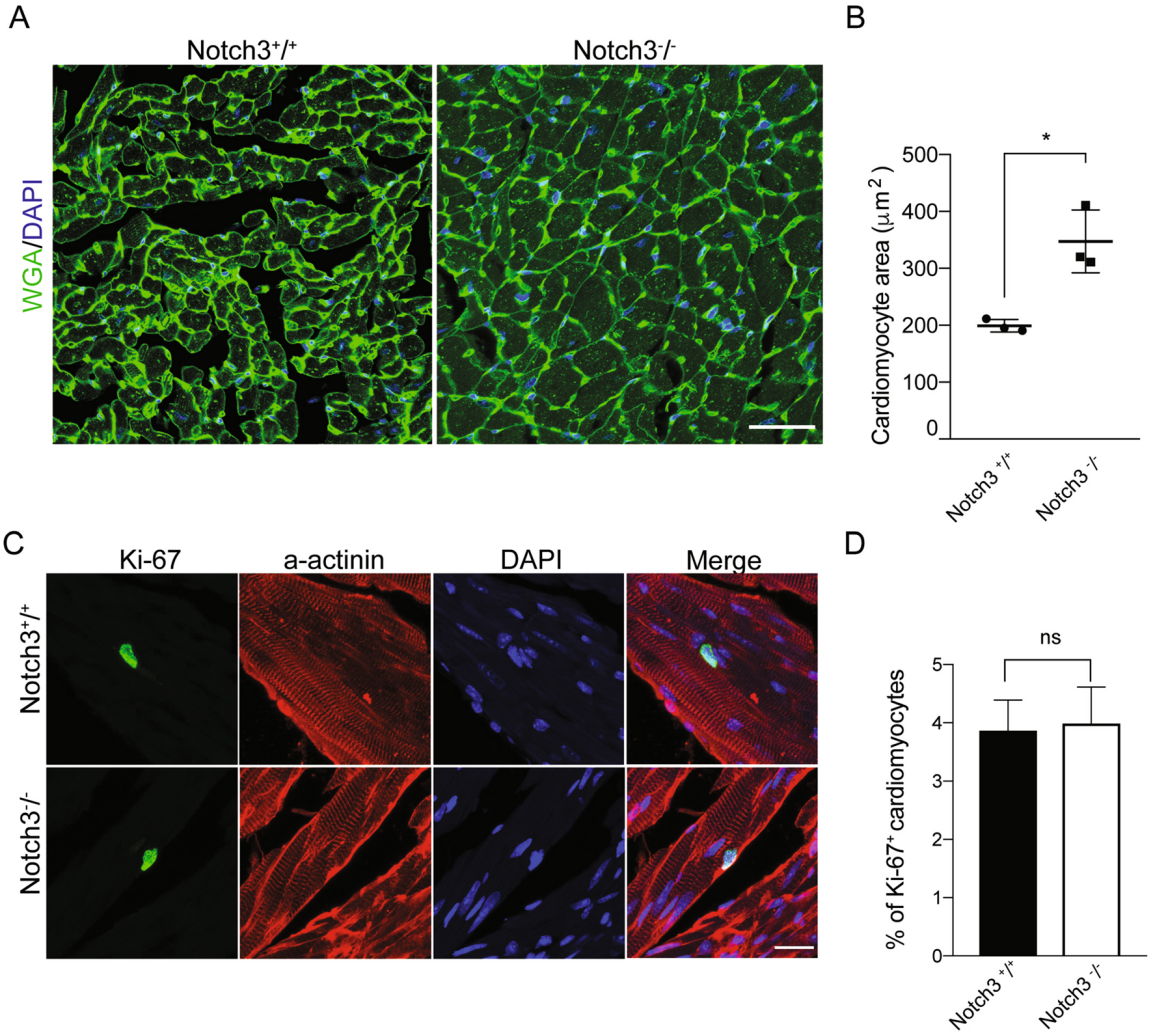
The left ventricular hypertrophy in the *Notch3*^*-/-*^ mice is associated with cardiomyocyte hypertrophy. (**A**) Wheat germ agglutinate (WGA) staining (green) and DAPI (blue) of heart cross-sections from control (*Notch3*^+*/*+^) and *Notch3*^*-/-*^ mice. Size bar = 50 μm. (**B**) Quantification of cardiomyocyte size (from A) n = 3 for each genotype. (**C**) Ki-67 staining of cardiomyocytes (with a-actinin as a marker for cardiomyocytes). Size bar = 20 μm. (**D**) Quantification of Ki-67 staining (from C). * p < 0.05, ns = non-significant. The mice used for these experiments were 10–14 months old.

The absolute majority of left ventricular hypertrophy is caused by cardiomyocyte hypertrophy, but there are also reports indicating that proliferation may be induced in postnatal cardiomyocytes in response to injury or perturbation of major signalling pathways^23,24^. To distinguish between these two possibilities, Wheat Germ Agglutinin staining, to outline the cardiomyocyte cell membranes, showed an increase in cardiomyocyte cross section area from 200 to 347 μm^2^ in the *Notch3*^*-/-*^ mice (Fig. 2A,B). Proliferation was assessed by Ki-67 immunostaining, and there was no increase in Ki-67 labelling in the *Notch3*^*-/-*^ mice (Fig. 2C,D).

An alternative cause of ventricular hypertrophy is hypertension^25^, but *Notch3*^*-/-*^ mice have a normal blood pressure unless challenged with angiotensin-II^21^. In line with a normal blood pressure, the expression of the *NppA* gene, which encodes atrial natriuretic peptide (ANP) and shows increased expression in response to hypertension^26^, was not upregulated (Supplemental Figure 1A). In sum, we conclude that the left ventricular hypertrophy is associated with an increase in cardiomyocyte size but not with increased cardiomyocyte proliferation.

### Cardiomyocyte morphology and marker expression are altered in the ***Notch3***^***-/-***^ mice

**Figure 3:**
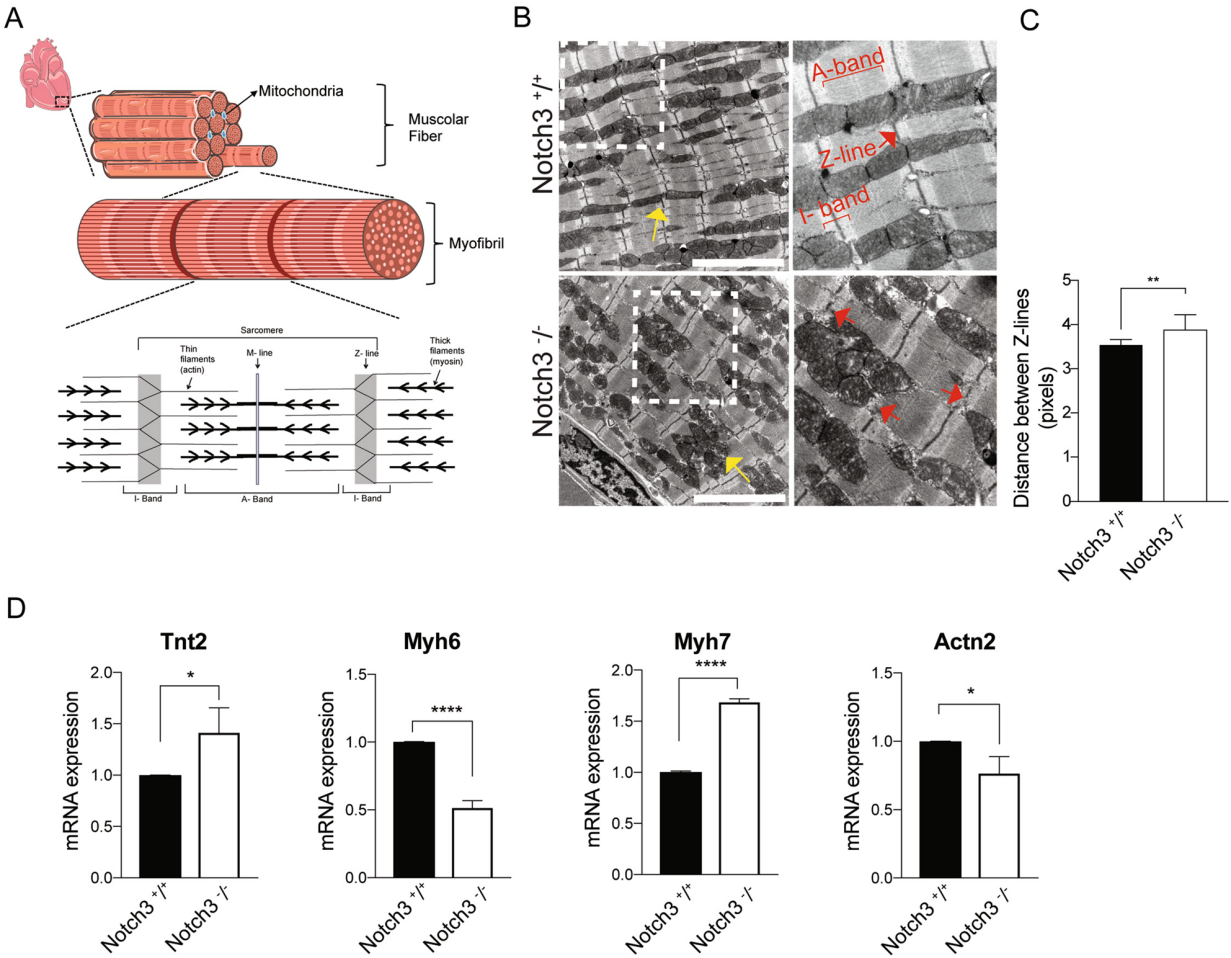
Altered sarcomere morphology and marker expression in the *Notch3*^*-/-*^ mice. (**A**) Schematic depiction of myofibrils and the organization of the sarcomere. The upper part of the figure was modified from a figure in Servier Medical Art, provided by Servier, licensed under a Creative Commons Attribution 3.0 unported license. (**B**) Transmission electron microscope (TEM) images from control (*Notch3*^+*/*+^) and *Notch3*^*-/-*^ hearts (12–13 months old). Yellow arrows indicate mitochondria while red arrows indicate Z-lines. A-bands and I-bands are also indicated in the figure. The white boxed area is enlarged in the high magnification views to the right. Size bar = 5μm. (**C**) Analysis of the distance between the Z-lines in *Notch3*^*-/-*^ sarcomeres. N = 2 animals per genotype (10–14 months old) were used and 9 fields of image per genotype were used for quantification. (**D**) Expression of *Tnt2*, *Myh6*, *Myh7* and *Actn2* mRNA from n = 4 control and n = 4 *Notch3*^*-/-*^ hearts. *p < 0.05, **p < 0.01, ****p < 0.0001.

Left ventricular hypertrophy may result from defects in the contractile capacity of the cardiomyocytes, for example because of mutations in sarcomeric genes^27–29^. The myocardium is built from cardiomyocytes, which harbour myofibrils. The myofibrils contain myosin and actin as well as auxiliary proteins such as tropomyosin and troponins and are built from aligned sarcomeres, a contractile apparatus which anchor to the α-actinin-containing Z-lines in the cardiomyocytes (Fig. 3A). Between the Z-lines at opposing ends of the sarcomere is the M-band, and titin filaments span between the M-band and the Z-lines. The A-band correspond to the myosin part, while the I-band corresponds to the actin filaments between the Z-lines and the A-band^27–29^ (Fig. 3A).

To learn whether the sarcomeric structure was altered in *Notch3*^*-/-*^ mice, we analysed sections from *Notch3*^*-/-*^ and control hearts by transmission electron microscopy (TEM). This revealed that the organization of mitochondrial tubules in linear arrays along the sarcomere was disturbed in *Notch3*^*-/-*^ hearts, which showed a looser adhesion between individual tubules (Fig. 3B). Furthermore, the I-bands were less pronounced (Fig. 3B) and the distance between the Z-lines was increased in the *Notch3*^*-/-*^ sarcomeres (Fig. 3C). In contrast, the distance between the A-bands was not statistically different in the *Notch3*^*-/-*^ sarcomeres (data not shown).

In line with an aberrant sarcomere structure, expression of genes encoding sarcomeric cardiomyocyte proteins was altered. Notably, expression of *TnT2*, which codes for cardiac muscle isoform of troponin T (TNNT2) regulating cardiac muscle contraction in response to Ca2 ^+ ^and mutated in cardiomyopathies^30^, was increased (Fig. 3D). *Myh7*, which encodes myosin heavy chain (MHC)-β and is one of the genes mutated in familial hypertrophic myocardiopathy^31^ was similarly expressed at higher levels, while the related *Myh6* gene, coding for myosin heavy chain (MHC)-α and in mice mostly expressed in the ventricles^32,33^, showed reduced expression (Fig. 3D). Expression of *Actn2* (α-actinin-2), which encodes the major component of the Z-disc and associated with hypertrophic myopathy^34^, was reduced in the *Notch3*-deficient mice (Fig. 3D). In conclusion, *Notch3*^*-/-*^ cardiomyocytes exhibit an aberrant morphology, accompanied by altered expression of genes involved in sarcomeric function.

### Dysregulated fatty acid and glycolytic metabolism in the ***Notch3***^***-/-***^ heart

**Figure 4:**
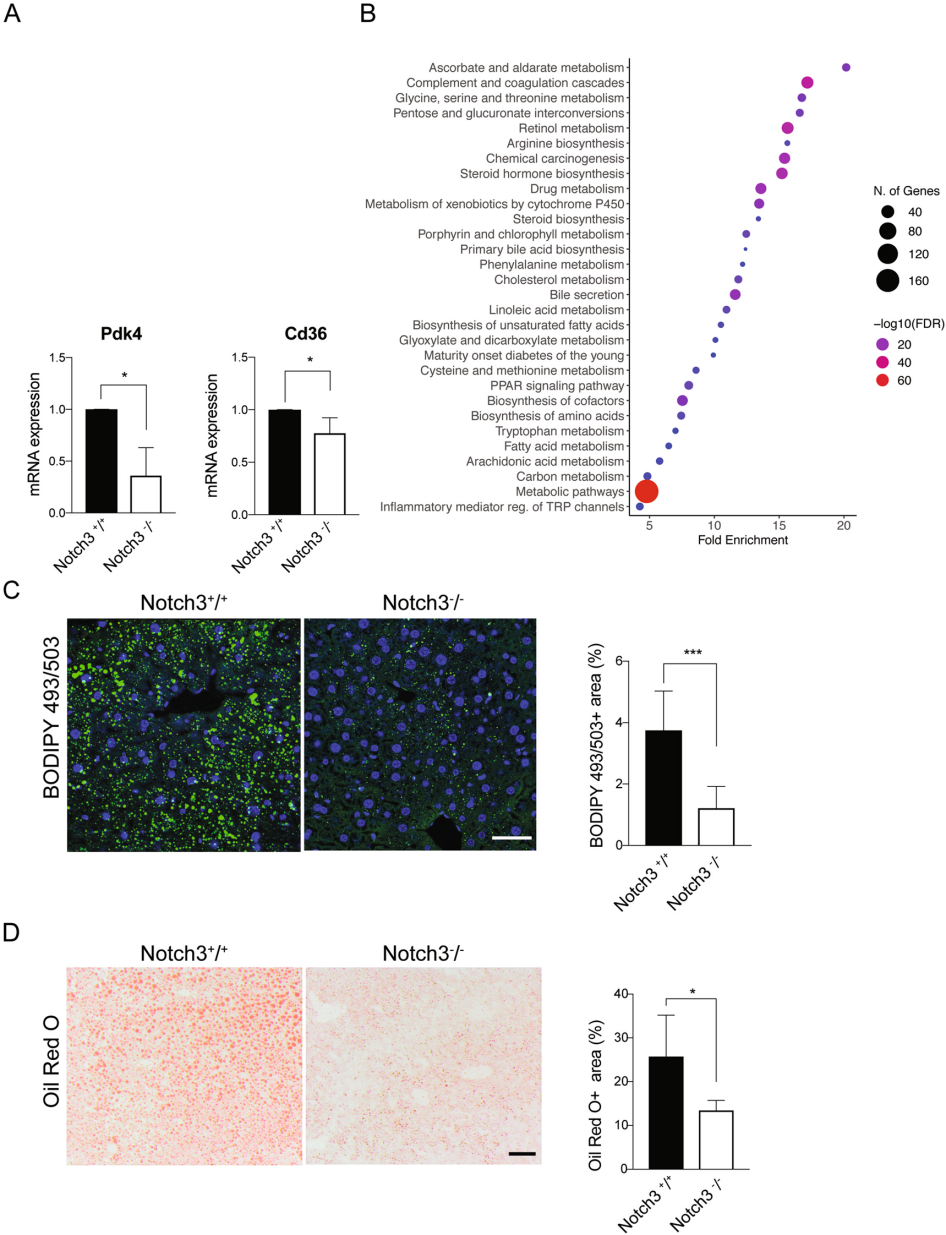
Dysregulated fatty acid and glucose metabolism in the *Notch3*^*-/-*^ heart. (**A**) Expression of the *Pdk4* and *Cd36* genes in n = 3 control (*Notch3*^+*/*+^) and n = 3 *Notch3*^*-/-*^ mice. (**B**) KEGG pathway analysis from bulk RNA-sequencing from hearts from n = 4 control and n = 3 *Notch3*^*-/-*^ mice (10–14 months old). (**C**) Left panel: Bodipy 493/503 staining of lipids in the livers of control and *Notch3*^*-/-*^ mice (6 months old). Size bar = 50μm. Right panel: quantification of the Bodipy results from B. N = 3 mice per genotype were used. (**D**) Left panel: Oil Red O staining of lipids in livers from control and *Notch3*^*-/-*^ mice (14 months old). Size bar = 200μm. Right panel: quantification of C. N = 4 mice per genotype were used. *p < 0.05, ***p < 0.001.

The adult heart relies largely on uptake of fatty acids to meet its energy demands, while the fetal heart preferentially uses glycolytic metabolism^35,36^; for review see^37^. Upon heart injury or disease, the adult heart however switches towards a more glycolytic metabolism^38^. *Pdk4*, which decreases glucose oxidation and increases fatty acid oxidation^39^, and *Cd36*, encoding the long chain free fatty acid transporter CD36^40^, were both downregulated in the *Notch3*^*-/-*^ hearts (Fig. 4A). Expression of peroxisome proliferation-activated receptor-gamma (PPARγ), which enhances glucose metabolism and causes insulin desensitization^41^, was slightly, although not significantly upregulated, while there was a tendency towards downregulation for expression of acyl-CoA dehydrogenase long and medium chain (*Acadl*, *Acadm*), which regulate beta oxidation of fatty acids^42^ (Supplemental Figure 1B). At the immunohistochemistry level, CD36 immunofluorescence was downregulated while the transporter for uptake of glucose (GLUT1) was upregulated in the adult *Notch3*^-/-^ hearts (Supplemental Figure 1C,D). In two-weeks old mice, there was no significant difference in CD36 and GLUT1 immunofluorescence (Supplemental Figure 1E), while CD36 immunofluorescence was decreased in the *Notch3*^*-/-*^ hearts at two months of age, accompanied by an increase in GLUT1 expression (Supplemental Figure 1F). At two months of age, there was also an increase in the cardiomyocyte area (Supplemental Figure 1G). We analysed whether expression of some of the Notch downstream genes important for the heart and vasculature was altered. Expression of the *Hey2* gene, which is important for heart development and is linked to Brugada syndrome, a rare form of arrhythmia^43,44^, was downregulated in the *Notch3*^*-/-*^ hearts, which as expected also was the case for *Notch3* (Supplemental Figure 1H). In contrast, *Hey1* expression was not affected (Supplemental Figure 1H).

To extend the gene expression analysis, we performed bulk transcriptomic analysis of *Notch3*^*-/-*^ and control hearts. Kyoto Encyclopedia of Genes and Genomes (KEGG) pathway analysis of differently expressed genes (DEG) in *Notch3*^*-/-*^ compared to control hearts (Supplemental Table 1) demonstrated that pathways related to for example metabolism and fatty acid degradation were dysregulated in *Notch3*^*-/-*^ hearts (Fig. 4B and Supplemental Table 2). In addition, we found that the DEG are enriched in diseases such as vascular diseases, lipid metabolism disorders and diseases related to liver dysfunction (Supplemental Figure 1 and Supplemental Table 3).

This shift in gene expression towards a more glycolytic profile could be caused by a direct more local effect on the cardiomyocytes mediated by the VSMC in the heart or be a result of a more general altered composition of nutrients in the blood. To address the latter hypothesis, we analysed whether the amount of lipid in the liver was altered, as an indicator of the lipid status in the vasculature. Staining with the lipid tracer BODIPY 493/503, which identifies neutral and non-polar lipids, revealed a reduction in lipid droplets in the *Notch3*^*-/-*^ liver (Fig. 4C), which was confirmed by Oil Red O staining (Fig. 4D). In sum, the data demonstrate a gene expression switch towards glycolytic metabolism in the *Notch3*^*-/-*^ hearts, accompanied by reduced lipid deposition in the *Notch3*^*-/-*^ livers.

### Notch3 is expressed in heart vascular smooth muscle cells but not in cardiomyocytes

**Figure 5:**
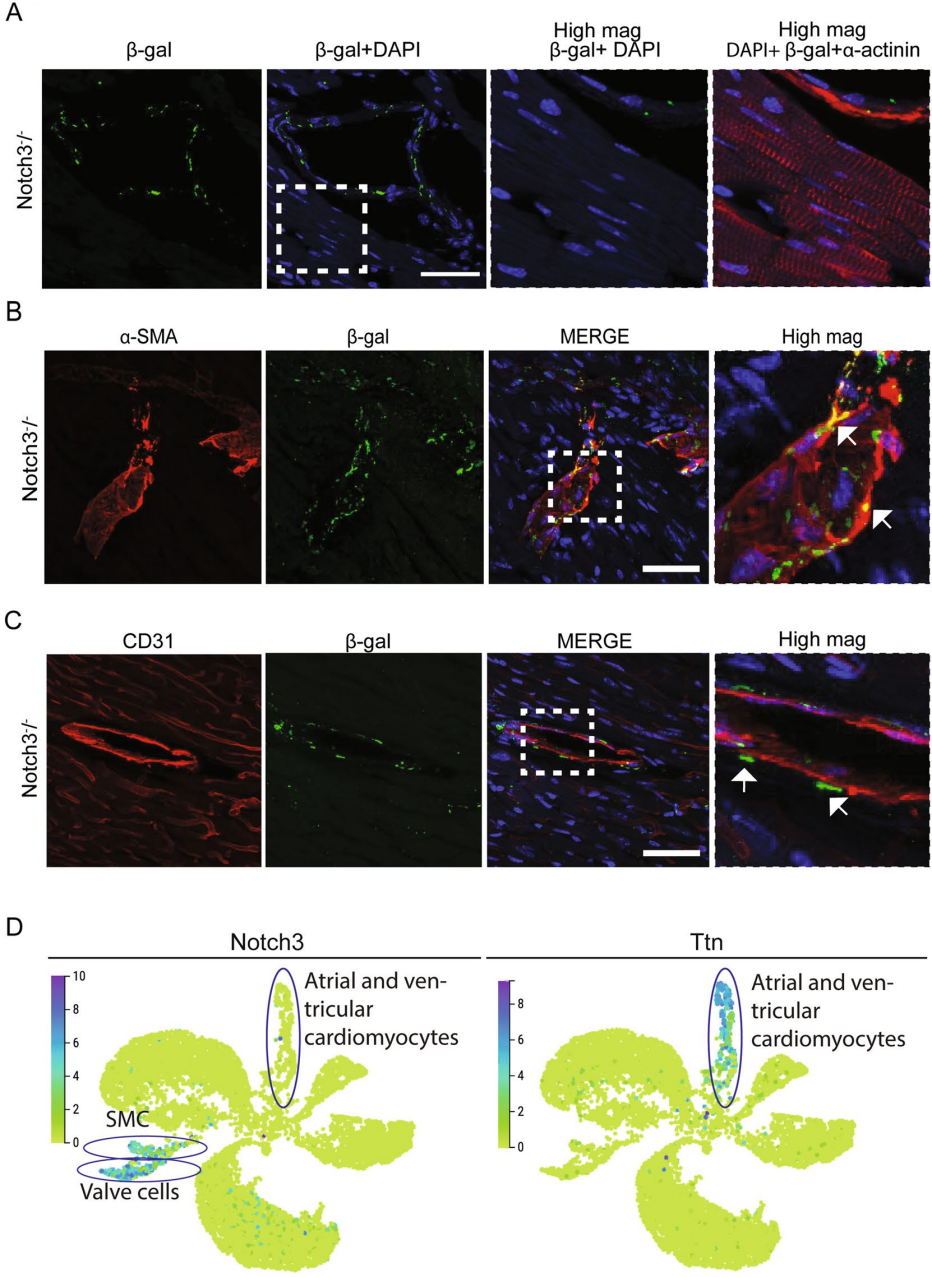
Notch3 expression in VSMC but not in cardiomyocytes. (**A**) Analysis of potential co-expression of beta-galactosidase (β-gal; as a proxy for Notch3) and α-actinin, a marker for cardiomyocytes, in sections from *Notch3*^*-/-*^ hearts (12–14 months old mice). The white boxed area is enlarged in the high magnification views to the right. Size bar = 50μm. (**B**) Analysis of co-expression of beta-galactosidase and αSMA as a marker for VSMC in *Notch3*^*-/-*^ heart sections. The arrows denote co-expression of beta-galactosidase and aSMA (appearing as yellow cells by the merged green and red signals). Size bar = 20μm. (**C**) Analysis of co-expression of beta-galactosidase and CD31 as a marker for endothelial cells in *Notch3*^*-/-*^ heart sections. The arrows denote beta-galactosidase-positive cells (green) adjacent to endothelial cells (red) in a blood vessel. Size bar = 50 μm. (**D**) Analysis of single cell transcriptomic data from adult mouse heart from the *Tabula Muris Senis* database (https://tabula-muris-senis.ds.czbiohub.org/). Expression of the *Notch3* (left) and *Titin* (*Ttn*) genes in UMAP projections of adult mouse heart cells is shown (high expression in purple/blue; low expression in green). The distributions of smooth muscle cells (SMC), valve cells and arterial and ventricular cardiomyocytes are encircled in the figure.

It is well established that *Notch3* is expressed in VSMC and pericytes in the vasculature^15,45,46^, but whether it also is expressed in cardiomyocytes has not been established, and to address this is important to understand whether the observed cardiomyocyte phenotype is a consequence of a cell-autonomous (loss of *Notch3* function in the cardiomyocytes) or non-autonomous (loss of *Notch3* function in mural cells) effect. To assess the expression of *Notch3* in the heart, we took advantage of a targeted insertion of the *lacZ* gene into the *Notch3* locus in the *Notch3*^*-/-*^ mice^47^, and *Notch3* expression can thus be monitored by beta-galactosidase immunohistochemistry as a proxy for *Notch3* expression^15^. In line with this notion, beta-galactosidase immunofluorescence was only observed in the *Notch3*^*-/-*^ mice, which harbour the targeted Notch3 alleles, and not in wildtype mice (Supplemental Figure 3). There was no expression of beta-galactosidase in cardiomyocytes (visualized by α-actinin as a marker for cardiomyocytes) (Fig. 5A), indicating that no or only very low levels of the Notch3 receptor is produced in cardiomyocytes.

In contrast to the lack of Notch3 expression in cardiomyocytes, Notch3 was expressed in the VSMC of the heart vasculature: co-staining for beta-galactosidase and the VSMC marker alpha-smooth muscle actin (αSMA; gene name *Acta2*) demonstrated extensive co-expression of Notch3 and αSMA at the rim of blood vessels (Fig. 5B). Furthermore, beta-galactosidase staining was adjacent to, but not overlapping with cells expressing CD31, a marker for endothelial cells (Fig. 5C), in keeping with expression in VSMC. To corroborate these findings, we next analysed the distribution of *Notch3* mRNA expression in different cell types in the Tabula Muris gene expression atlas^48^. *Notch3* expression was predominantly observed in smooth muscle cells and valve cells, while expression was negligible in cardiomyocytes (Fig. 5D). As a control, expression of *Ttn* (Titin), a cardiomyocyte marker, was largely confined to cardiomyocytes (Fig. 5D).

**Figure 6:**
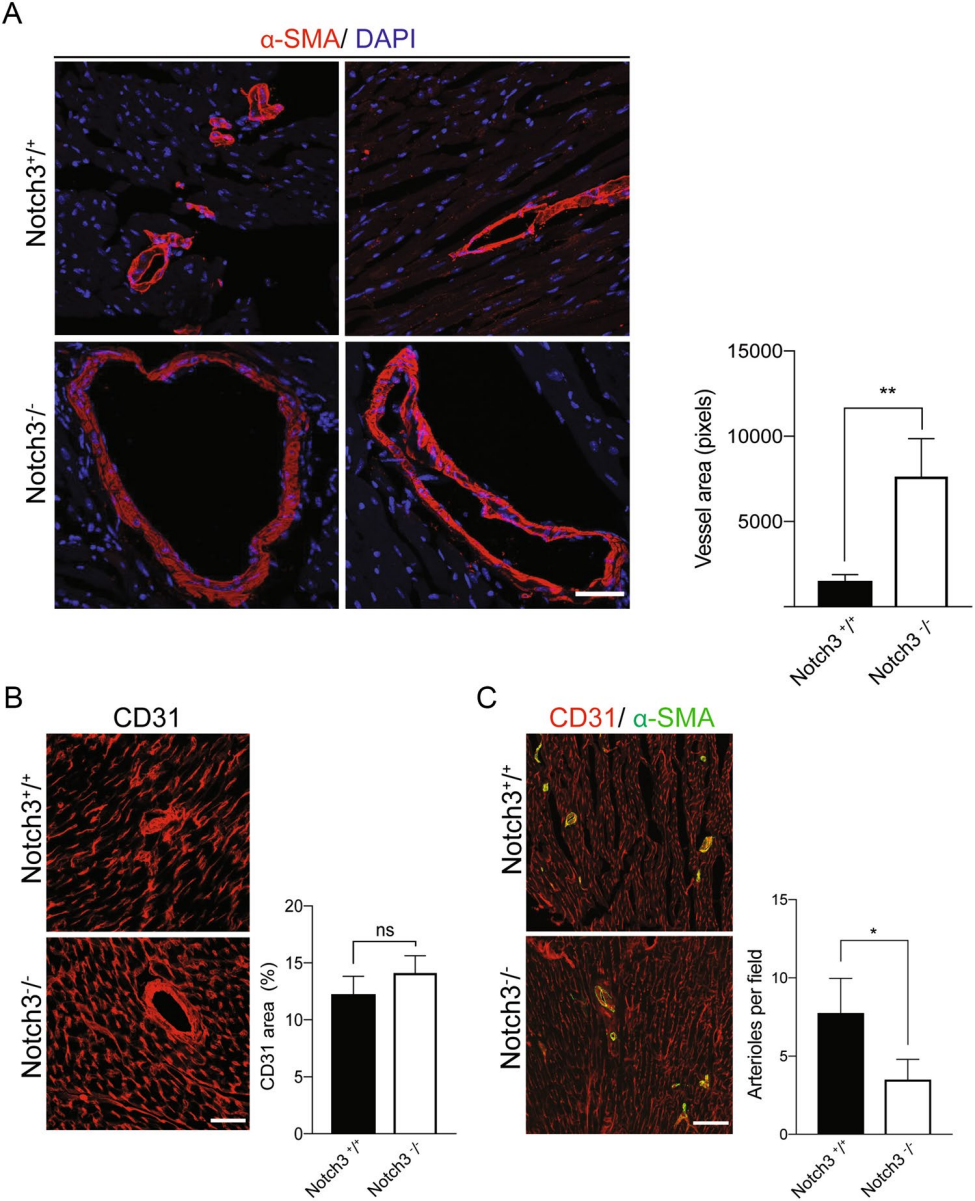
*Notch3*-deficiency leads to structural changes in the vasculature. (**A**) Examples of vessel dilation in the *Notch3*^*-/-*^ heart. Size bar = 50 μm. (**B**) CD31-staining in sections from the hearts of control and *Notch3*^*-/-*^ mice. To the right, the quantification of the CD31-staining area is presented. N = 3 mice and a minimum of 2 fields per image for each mouse were used for quantification. Size bar = 100 μm. (**C**) Analysis of αSMA (marking arteriolar VSMC) and CD31 (marking endothelial cell) staining in sections from the hearts of control (*Notch3*^+*/*+^) and *Notch3*^*-/-*^ mice. To the right, the quantification of arterioles per field is presented. N = 3 mice and a minimum of 2 fields per image for each mouse were used for quantification. The mice used for these experiments were 10–14 months old. Size bar = 100 μm. *p < 0.05, **p < 0.01, ns = non-significant.

As *Notch3*-deficiency can lead to structural changes in the vasculature^15^, we next assessed whether blood vessel morphology was altered in the *Notch3*^*-/-*^ hearts. Several vessels were more dilated in the *Notch3*^*-/-*^ hearts (Fig. 6A), while there was no difference in the density of the vasculature, as judged by CD31 expression per area (Fig. 6B). The number of arterioles per area was however reduced in the *Notch3*^*-/-*^ hearts (Fig. 6C). In conclusion, *Notch3* is expressed in VSMC but not cardiomyocytes in the heart, and loss of Notch3 is associated with morphological changes in the cardiac vasculature.

### ***Pdgfb***^***ret/ret***^ mice exhibit left ventricular hypertrophy

**Figure 7:**
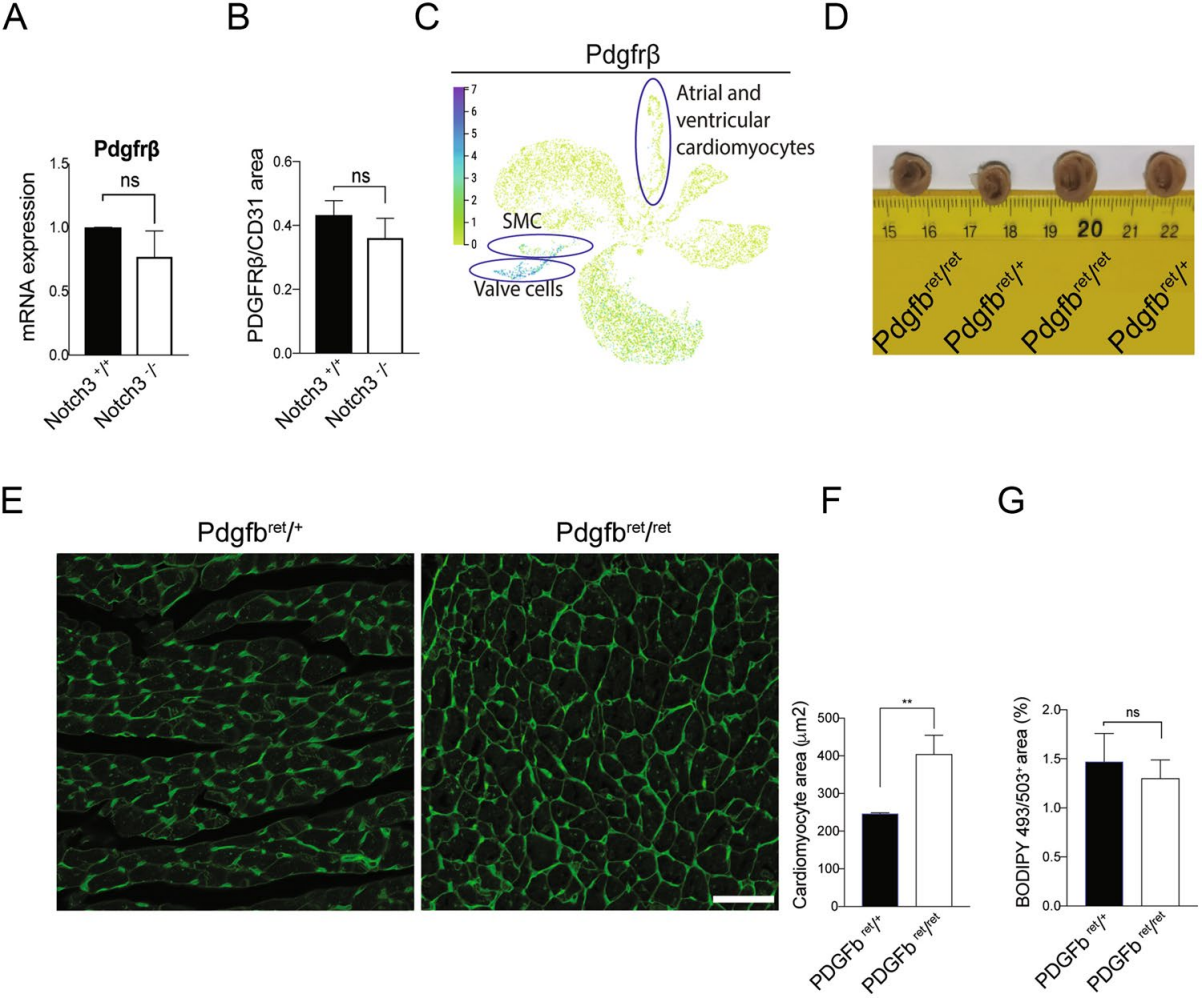
Reduced PDGF signalling causes left ventricular hypertrophy. (**A**) Expression of the Notch downstream gene *Pdgfrb*. N = 3 mice per genotype were analysed. (**B**) Immunohistochemistry analysis of PDGFRb staining over CD31 area. N = 3 mice per genotype were analysed. (**C**) Analysis of single cell transcriptomic data from adult mouse heart from the *Tabula Muris Senis* database (https://tabula-muris-senis.ds.czbiohub.org/). Expression of the *Pdgfrb* gene in heart tissue of adult wildtype mice. (**D**) The size of the hearts in control (*Pdgfb*^*ret/*+^) and *Pdgfb*^*ret/ret*^ mice. (**E**) WGA staining of heart cross-sections from control and *Pdgfb*^*ret/ret*^ mice. (**F**) Quantification of cardiomyocyte size (from **E**). N = 3 mice per genotype were analysed. (**G**) Quantification of the Bodipy 493/503 results from *Pdgfb*^*ret/ret*^ mice. N = 3 mice per genotype were analysed. Size bar = 50 μm. The mice used for these experiments were 8 months old. **p < 0.01, ns = non-significant.

*Platelet-derived growth factor receptor beta (Pdgfrb)* is important for pericyte function^49^. *Pdgfrb* is also a Notch downstream target gene^12,50^ and there were trends towards downregulation of both *Pdgfrb* mRNA and protein expression in *Notch3*^*-/-*^ hearts (Fig. 7A,B). As *Pdgfrb*, like *Notch3*, was expressed in SMC and valve cells in the heart but not in cardiomyocytes (Fig. 7C), we decided to explore whether perturbed signalling via the PDGFRB receptor led to a heart phenotype. To this end, we used the PDGFB retention (*Pdgfb*^*ret/ret*^) mouse model, which produces a truncated PDGFB ligand^51^ and has been extensively used as a tool to explore perturbed PDGF signalling in the vasculature^49,52^. Like the *Notch3*^*-/-*^ mice, the *Pdgfb*^*ret/ret*^ mice exhibited enlarged hearts (Fig. 7D), accompanied by left ventricular hypertrophy (Fig. 7E,F). The *Pdgfb*^*ret/ret*^ mice however differed from the *Notch3*^*-/-*^ mice in terms of lipid accumulation in the liver; the BODIPY 493/503 staining area was not altered in the *Pdgfb*^*ret/ret*^ mice (Fig. 7G). In conclusion, the *Pdgfb*^*ret/ret*^ mouse exhibits left ventricular hypertrophy similar to what is observed in the *Notch3*^*-/-*^ mice, but the two mouse models differ with regard to lipid accumulation in the liver.

## Discussion

In this report, we show that *Notch3*^*-/-*^ mice, in addition to the well-established progressive loss of VSMC in the vasculature^15^, present a heart phenotype: left ventricular hypertrophy, coupled with cardiomyocyte hypertrophy and mild fibrosis. *Notch3* was expressed in VSMC and pericytes of the heart but not in cardiomyocytes, leading us to propose that the effects on the heart are indirect and a consequence of the loss of Notch3 function in the VSMC in the vasculature of the heart or elsewhere in the body. The left ventricular hypertrophy in the *Notch3*^*-/-*^ mice was more profound compared to ablation of CSL in VSMC postnatally^21^, suggesting that the onset of *Notch3*^*-/-*^ hypertrophy may occur at an earlier developmental stage. In keeping with this notion, we observed an increase in cardiomyocyte area and dysregulated CD36 and GLUT1 expression already at two months of age in the *Notch3*^*-/-*^ mice.

To gain insights into the *Notch3*^*-/-*^ heart phenotype, we analysed the expression of genes important for cardiac metabolism, and expression of *PDK4*, which is involved in fatty acid uptake, and *GLUT1*, responsible for glycolysis, was down- and up-regulated, respectively. This may indicate that the *Notch3*^*-/-*^ heart switches from oxidative phosphorylation towards a more glycolytic metabolism. The adult heart uses mostly fatty acids as energy source, with fatty acids from the blood transported through endothelial cells into the heart^53^. However, when diseased or injured, the heart reverts to a glycolytic metabolism, which is otherwise observed at embryonic stages^35,36^; for review see^37^. The observed switch towards a glycolytic metabolism in the *Notch3*^*-/-*^ hearts may therefore be a consequence of the left ventricular hypertrophy. Alternatively, *Notch3* deletion and the subsequent loss of VSMC may in some way lead to a more systemic lowering of lipid levels in the vasculature, forcing a switch to higher glucose uptake and a glycolytic metabolism. The latter notion is supported by the lower lipid content in the livers of *Notch3*^*-/-*^ mice. It is also interesting to note that when Notch signalling was abrogated in the endothelium through deletion of the CSL gene in endothelial cells, this resulted in left ventricular hypertrophy and metabolic remodelling of the heart with disturbed fatty acid transport across the vessel wall and higher glucose uptake^54^. An intriguing possibility is therefore that some of the secondary effects from loss of Notch3 function in the VSMC occur in the nearby endothelial cells, leading to reduced uptake of fatty acids, but to address this idea will require further experimentation.

Conceptually, how dysregulated Notch signalling in mural cells affects cardiomyocyte function could either be explained by specific effects on vasculature in the heart or by a more systemic effect on the arterial tree. In support of a first “cardio autonomous” model, we noted that the number of arterioles per area was reduced in the *Notch3*^*-/-*^ hearts, in keeping with two previous reports using a different Notch3 knockout model^21,55^, while the overall density of the vasculature was not changed. Interestingly, ablation of CSL in endothelial cells in contrast resulted in an increased density of the heart vasculature^54^. Whether the decrease in number of arterioles in the *Notch3*^*-/-*^ hearts contributes to the left ventricular hypertrophy remains to be explored. Alternatively, a “non-cardio autonomous” model would posit that more global changes to the vasculature would lead to systemic effects, presumably through hemodynamic mechanisms, thereby negatively impacting cardiomyocytes of the heart. The best-known example of this would be left ventricular hypertrophy resulting from chronic hypertension^25^, which is unlikely the cause of left ventricular hypertrophy in *Notch3*^*-/-*^ mice, as they have a normal blood pressure unless subjected to angiotensin-II^21^. *Notch3*^*-/-*^ mice have however been shown to exhibit deficiencies in the vascular tone of cerebral, renal, and tail resistance arteries^56,57^, and it is possible that a more general defect in VSMC contractility in resistance arteries would require compensatory hemodynamic mechanisms of the heart to overcome the chronic pressure overload in the *Notch3*^*-/-*^ mice. A compensatory increase in heart rate and/or stroke volume could contribute to the cardiac remodeling seen in the *Notch3*^*-/-*^ mice and would be in line with the observed increase in the interval between Z-lines in the sarcomeres and elevated *Nppb* expression in the *Notch3*^*-/-*^ mice. Further studies are however required to understand whether local and/or systemic effects on the vasculature contribute to molecular and morphological changes in *Notch3*-deficient hearts. Irrespective of the nature of the link between perturbed vascular Notch signalling and a heart phenotype, an indirect effect from the vasculature on the cardiomyocytes may explain why heart problems were observed as a side effect of clinical trials using blocking antibodies to Dll4, a Notch ligand expressed predominantly in endothelial cells^58,59^.

The notion, based on analysis of the *Notch3*^*-/-*^ mice, that impairment of the heart can be an indirect result of genetic perturbations primarily the vasculature was corroborated by observations from the *Pdgfb*^*ret/ret*^ mice. The *Pdgfb*^*ret/ret*^ mice have reduced PDGF signalling because the truncated PDGF-B ligand lacking the retention motif is more diffusible, leading to lower local PDGF-B concentration around PDGF-B producing endothelial cells^51^ and, consequently, less stimulation of neighbouring PDGFRβ–positive VSMC and pericytes. It is noteworthy that the heart phenotype in the *Pdgfb*^*ret/ret*^ mice was partly similar to the *Notch3*^*-/-*^ phenotype, i.e., both models exhibit left ventricular and cardiomyocyte hypertrophy, although the *Pdgfb*^*ret/ret*^ mice did not show a reduced lipid content in the liver. The similarity in heart phenotypes underscores that primary effects in mural cells can indirectly affect cardiomyocytes, and it is also possible that some of the effects observed in the *Notch3*^*-/-*^ mice may in fact be attributed to reduced *Pdgfrb* expression, as a small decrease in *Pdgfrb* mRNA and protein expression was noted in the *Notch3*^*-/-*^ mice. In conclusion, we provide evidence from analysis of mouse models with disturbed Notch and PDGF signalling that a primary perturbation in VSMC and pericytes is sufficient to cause a heart phenotype. To gain further insights into the interplay between the VSMC and pericytes and cardiomyocyte function will be important to explore new therapeutic strategies for heart disease caused by primary vascular impairment.

## Methods

### Mice

The *Notch3*^*-/-*^ mice (C57BL/6J background) were maintained at the Department of Comparative Medicine (KMA), Karolinska Institutet (Stockholm, Sweden). The *Pdgfb*^*ret/ret*^ mice were maintained at the Rudbeck Laboratory, Uppsala University, Sweden. The mice were provided with water and food ad libitum, were maintained in a 12 h light/dark cycle, and housed in enriched cages. All experimental animal procedures were performed in accordance with local regulations and rules and according to ARRIVE guidelines. All animal experiments were approved by the Stockholm’s North (the *Notch3*^*-/-*^ mice) and Uppsala (the *Pdgfb*^*ret/ret*^ mice) Ethical Committees for Animal Research (Ethical permit No 5253–2019 and 5.8.18–03,029/2020 respectively). Mice used in this study were 6–14 months old as indicated in the figure legends, except for Supplementary Fig. 1E-G, where 2 weeks and 2 months old mice were used.

### Histology

For the hematoxylin and eosin (HE) staining, mice were anesthetized and transcardially perfused with Hanks buffered salt solution (HBSS) followed by a solution of 4% formaldehyde in phosphate buffered saline (PBS). Tissues were post fixed in 4% formaldehyde for 4 h at 4 °C and transferred to 70% ethanol solution. After sectioning (4 μm sections), HE staining was performed as follows: The slides were baked for 40–60 min at 60 °C followed by deparaffinization in Xylene (Solveco) for 5 min (2 times) and rehydrated in absolute ethanol for 3–5 min (2 times), 95% ethanol for 2 min, 70% ethanol for 2 min. The slides were then rinsed with warm distilled running water for 2 min. The nuclear staining was performed using Mayer Hematoxylin (Histolab) for 10 min at room temperature (RT). The slides were then rinsed with warm distilled running water for 10–15 min followed by the cytoplasm staining (Eosin, Histolab) for 3 min at RT. After wash with warm distilled running water for 10–15 min, rehydration was performed (70% ethanol: 30 s, 95% ethanol: 2 min, Absolute ethanol: 3-5min for two times followed by two steps in Xylene for 5 min each. The slides were mounted with Pertex (Histolab).

For the Oil Red O staining, mice were euthanized, the organs removed and immediately fresh frozen and embedded in OCT (Scigene). 10 μm cryosections were left at RT for 15 min. Slides then were fixed with 4% PFA (Solveco) for 1 min. The slides were then rinsed with running tap water for 2 min followed by ORO staining (Histolab) for 3 min at RT. After 1 wash with running tap water for 2 min, slides were mounted with aqueous mounting medium (Mount it, Vector).

For the Masson’s staining, embedded paraffin blocks were sectioned to 4µm sections and Modified Masson's Trichrome Stain Kit (Sigma Aldrich) was applied according to the manufacturer’s instructions. Images of HE and Oil Red O staining and Masson’s staining were taken with the Olympus IX73 Microscope.

### Immunohistochemistry

Adult mice of both sexes (8–10 months) were euthanised and the heart, lung and liver were removed. Organs were postfixed with 4% buffered formaldehyde (Histolab Products AB) for 4 h at RT, washed in PBS and immersed in 30% sucrose/PBS solution until sinking. Organs were mounted for cryosectioning in OCT (Scigen). Cryosections (14µm) were stored at− 80 °C until further use. Cryosections were thawed at RT for 15 min then washed quickly in PBS, dried and a hydrophobic barrier was created using ImmedgeTM Hydrophobic Barrier Pen (Vector Laboratory, United States). Sections were then permeabilised in 0,3% Triton X for 10 min, washed in PBS and blocked for one hour at RT with Serum-Free Protein Block (Dako). Next, the sections were incubated with primary antibody solution at 4°C overnight, followed by 3 washes in PBS and finally secondary antibody solution for one hour at RT. For nuclear staining, sections were incubated with DAPI (Invitrogen) before mounting in ProLong Gold Antifade (Life Technologies). Antibodies were diluted in 0.5% bovine serum albumin, 0.1% Triton X-100 in PBS. All primary and secondary antibodies are described in Supplemental Table 4. Specimens were analysed using a Leica TCS SP8 confocal microscope (Leica Microsystems). All confocal images are represented as maximum intensity projections unless stated otherwise. Image analysis was carried out using Image J (NIH) software.

### Wheat germ agglutinin staining

Wheat germ agglutinin staining (WGA) was carried out as follows: Heart tissue was harvested from the mouse and post fixed with 4% buffered formaldehyde (Histolab Products AB) for 4 h at 4°C. The tissue was then immersed in a 30% sucrose solution at 4 °C until sinking. Subsequently, the tissue was embedded in OCT (Scigen) and stored at − 80 °C in Tissue-Tek cryomold (Sakura) until use. Frozen blocks were then cut into 10 μm sections and stored at -80 °C. Frozen sections were allowed to equilibrate at RT for 15 min and then washed in PBS for 5 min. The 1.0 mg/mL WGA conjugate (W11261, Thermo Fisher) stock solution was diluted into 20ug/ml with PBS and heart sections were incubated with WGA for 20 min, at RT in the dark. After 3 washes in PBS, the heart sections were incubated with DAPI (Invitrogen) for 5 min, at RT in darkness and washed in PBS for 5 min at RT. The sections were mounted with Prolong Gold antifade reagent (Invitrogen) and kept at 4 °C in darkness until visualisation with confocal microscopy (see above).

### Transmission electron microscopy

The heart tissue was fixed by perfusion using 3% glutaraldehyde and 1% formaldehyde in 0.1M phosphate buffer, pH 7.4. After perfusion, the heart tissue was harvested and allowed to fix at RT prior to storage at + 4 °C. Following fixation, the tissue was washed in 0.1M phosphate buffer and postfixed in 2% osmium tetroxide in 0.1M phosphate buffer at RT for 2 h before stepwise dehydrated in ethanol followed by acetone and finally resin-embedded in LX-112 (Ladd). Ultrathin sections (approximately 80–100 nm) were cut using an EM UC7 ultramicrotome (Leica) and contrasted with uranyl acetate followed by lead citrate. The sections were examined in a Hitachi HT7700 transmission electron microscope (Hitachi High-Technologies) operated at 80 kV and 2xk2x digital images were acquired using a Veleta CCD camera (Olympus Soft Imaging Solutions).

### qPCR analysis

Mice were euthanized, the organs removed and immediately fresh frozen. Frozen tissue from the left ventricle was cut on ice and homogenized in 0.5 ml Trizol (Thermo Fisher Scientific) per 25 mg tissue. Metallic beads are added to the mixture and TissueLyzer (Qiagen) was used with the following setting: 50Hz for 2 min twice with a fast spin in between. 0.1 ml of chloroform per 0.5 ml of Trizol was added, samples were shaken vigorously for 15 s and kept for 10 min at RT and then centrifuged for 5 min at 12,000 × g. The transparent aqueous phase was then transferred to a fresh tube. The RNA was precipitated with 250 μl of isopropanol per 0.5 ml of Trizol used for the initial homogenization. Samples were kept on ice for 1 h and centrifuged for 10 min at 4 °C at 12,000 × g. The pellet was washed once with 75% ethanol by flipping the tube without disturbing the pellet and subsequently centrifuged for 10 min at 12,000 × g (4 °C). The ethanol was removed, and the pellet resuspended in 50 μl of RNAse and DNAse free water (Ambion). RNA was converted to cDNA using the iScript Kit (Biorad) and the qPCR mixture was prepared using the SsoAdvanced Universal SYBR Green Supermix (Biorad). The qPCR primer list is provided in the Supplemental Table 5. qPCR was performed using the Applied Biosystems 7500 Real-Time PCR System (ABI 7500; Applied Biosystems). Relative gene expression was determined using the ΔΔCT method (ΔΔCT = ΔCt sample-Δct control) and fold change expression changes were calculated by normalizing first to *GAPDH* mRNA expression levels, as a housekeeping gene reference. A minimum of three biological replicates (three different animals per genotype) were used. All the graphs and the statistical analysis were produced using Prism Graphpad (ver.8) and differences in mRNA expression levels between samples were analysed using unpaired t-test.

### RNA-seq

The RNA was quantified with Qbit (Invitrogen) and loaded in a 384 well plate for subsequent cDNA conversion using a previously described protocol^60^. Briefly, the RNA undergoes a first-strand conversion in a reaction mixture with SuperScript II reverse transcriptase (Invitrogen), RNase inhibitor (Takara), 5X SuperScript II First-Strand buffer (Invitrogen), DTT (Invitrogen), 5M Betaine (Sigma) MgCl2 (Sigma), and custom locked nucleic acid oligonucleotides. Reverse transcription was performed for 10 cycles followed by inactivation at 70°C for 15 min. The total volume of cDNA was added into PCR master mix containing KAPA HiFi HotStart ReadyMix (KAPA Biosystems) and ISPCR primers. The PCR reaction was performed for 22 cycles. Next, PCR products were purified using SeraMag beads (Sigma Aldrich) and 17% PEG, with the final elution in EB buffer (Qiagen). The library quality and size distribution were checked using a High-Sensitivity DNA chip (Agilent Bioanalyzer). For the library preparation, cDNA samples were mixed with 10mM TAPS-NaOH, 5mM MgCl_2_ pH 8,5, 10% PEG 8K, Tn5 enzyme and water and incubated at 55°C for 6 min. After this step the samples were incubated with 0,2% SDS for 5 min at RT. Then the samples were pooled after enrichment PCR as described by the manufacturer (Illumina) through the use of a dual-index strategy, referred to as index 1 (i7) and index 2 (i5). The sequencing was then performed using a HiSeq 3000 unit at the Single Cell Core Facility (SICOF) at Karolinska Institute (Sweden).

### Bioinformatic analysis

Four and three biological samples of Notch3^+/+^ and Notch3^-/-^ mice, respectively, have been considered for differential gene expression analysis (DEG) and three technical replicates per biological sample were collapsed. DEG analysis was performed using the DESeq2 package version 1.41.4^61^. Differentially expressed genes between *Notch3*^*-/-*^ and control (*Notch3*^+*/*+^) mice with statistical significance were then identified with a minimum fold change 1.5 and a false discovery rate (FDR) lower than 0.05. Shrunken log fold changes were generated using the adaptive shrinkage estimator ashr^62^. For the full list of differentially expressed genes (DEG), see Supplemental Table 1. KEGG pathway enrichment and Jensens disease analysis of the differentially expressed genes were carried out using ShinyGO v0.77 (http://bioinformatics.sdstate.edu/go/) with a FDR cutoff of 0.05 and a minimum pathway size of 10^64^. Data tables with pathway enrichment values, gene information and GO terms were downloaded directly from the website.

### Image analysis

For all histology, immunofluorescence and area images (Figs. 1D, 1F, 2A, 4B, 4C, 6B, 6C, 7F, 7G) a minimum of two/ three animals per genotype were used and three/four pictures were randomly taken and analysed. Briefly, the colour channel was split and converted into 8-bit greyscale in Image J (NIH) and subjected to automatic threshold using the Li method. Regions of interest were drawn and % threshold value of the indicated marker was used (Figs. 4B, 4C, 6B, 6C, 7G). For the cardiomyocyte area measurement, the cells per field were counted and the cross area was measured with ImageJ software (Figs. 2A, 7F). For Fig. 1F, random measurements of the left ventricle wall were made. All the graphs and the statistical analysis were performed using GraphPad Prism (ver.8) and unpaired t-test.

#### Analysis of Tabula Muris

The data in Figs. 5D and 7G were obtained from https://tabula-muris-senis.ds.czbiohub.org/ by selecting the FACS method for *heart* tissue and searching for the gene of interest.

### Supplementary Information

Supplementary Figures.

Supplementary Table 1.

Supplementary Table 2.

Supplementary Table 3.

Supplementary Table 4.

Supplementary Table 5.

## Data Availability

The transcriptomic data have been uploaded at Gene Expression Omnibus (GEO) with Accession Number GSE237040.
