# Supplementary material for: Left ventricular hypertrophy and metabolic resetting in the Notch3-deficient adult mouse heart
Source: Sci Rep. 2023 Sep 12;13:15022. doi: 10.1038/s41598-023-42010-7 (PMC10497627; doi:10.1038/s41598-023-42010-7)
Supplement: Supplementary file 1 — Supplementary Figures. [file 41598_2023_42010_MOESM1_ESM.pdf]

Supplemental Information

Left ventricular hypertrophy and metabolic resetting in the *Notch3*-deficient adult mouse heart

Francesca Del Gaudio, Dongli Liu, Maarja Andaloussi Mäe, Eike-Benjamin Braune, Emil M. Hansson, Qing-Dong Wang, Christer Betsholtz, Urban Lendahl

Supplemental Figure 1

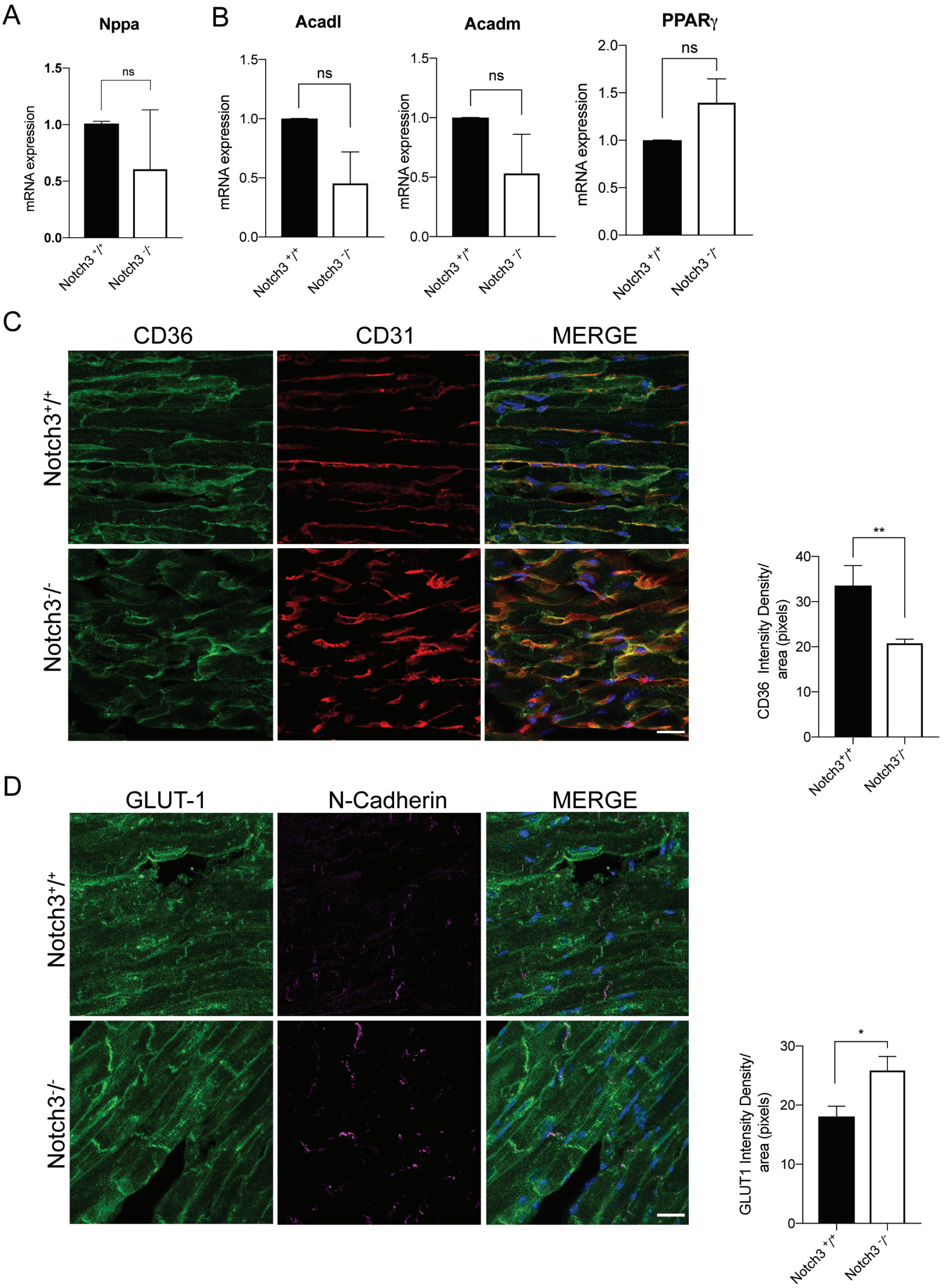

E

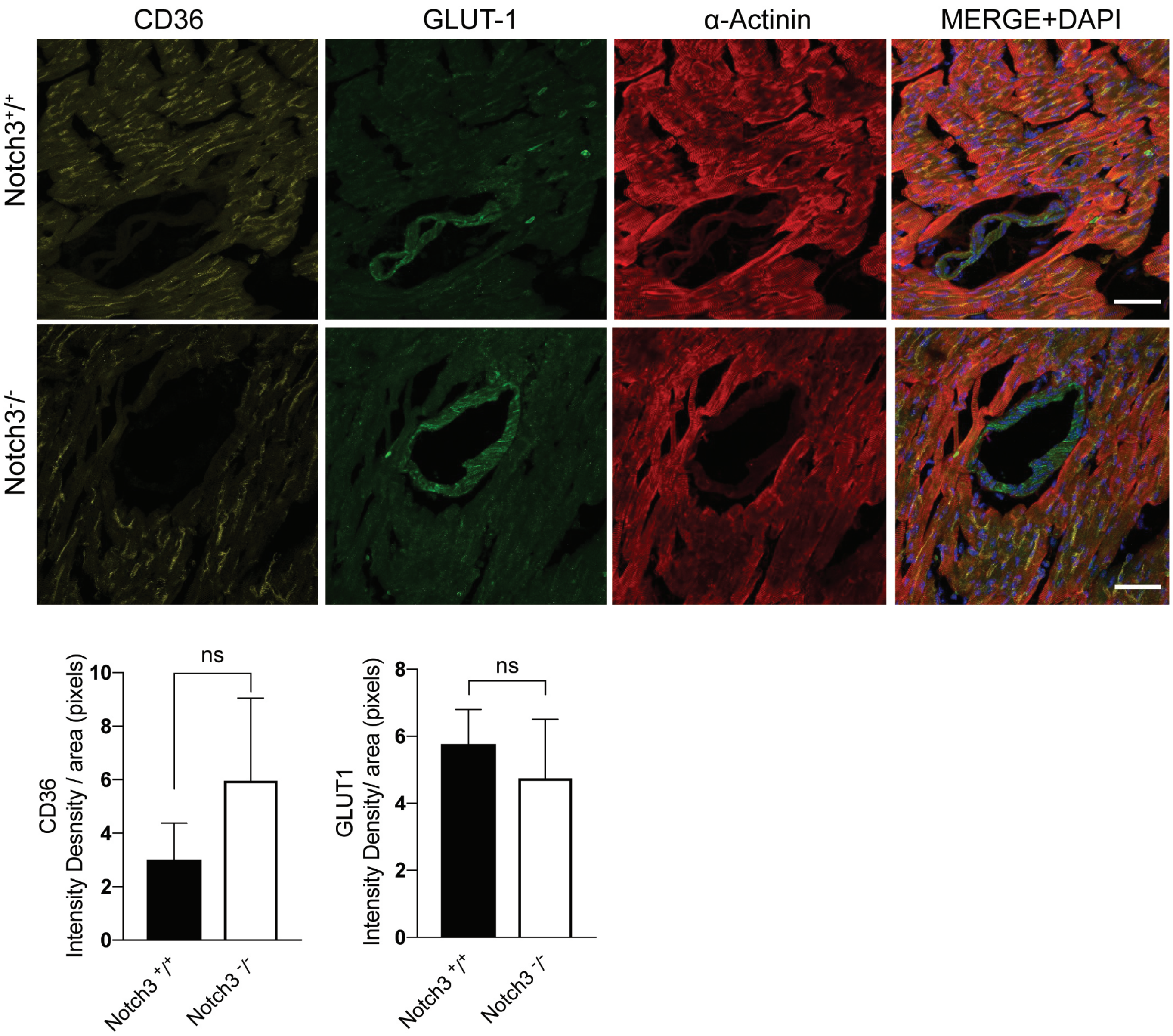

CD36
GLUT-1
α-Actinin
MERGE+DAPI
Notch3+/+
Notch3-/-
ns
CD36
Intensity Desnsity / area (pixels)
10
8
6
4
2
0
Notch3 +/+
Notch3 -/-
ns
GLUT1
Intensity Density/ area (pixels)
8
6
4
2
0
Notch3 +/+
Notch3 -/-

F

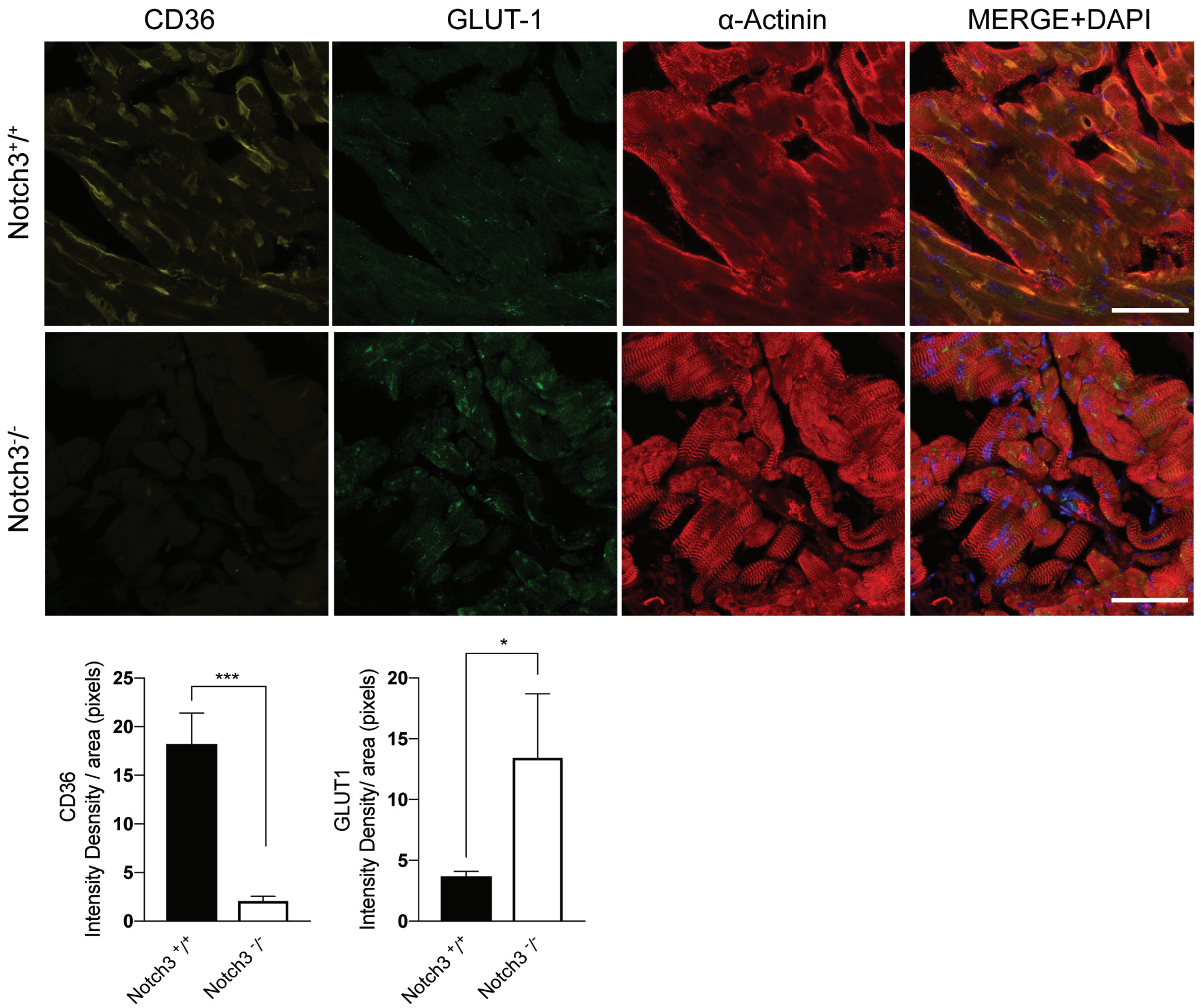
CD36
GLUT-1
α-Actinin
MERGE+DAPI
Notch3+/+
Notch3-/-
CD36
Intensity Desnsity / area (pixels)
25
20
15
10
5
0
***
Notch3 +/+
Notch3 -/-
GLUT1
Intensity Density/ area (pixels)
20
15
10
5
0
*
Notch3 +/+
Notch3 -/-

G

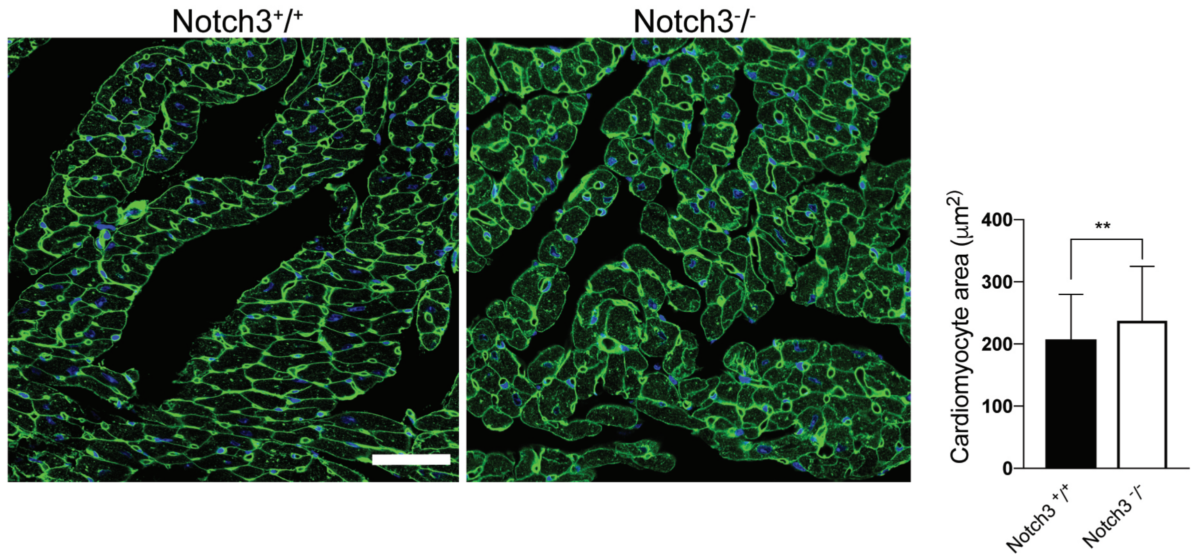
Notch3+/+
Notch3-/-
Cardiomyocyte area (μm2)
400
300
200
100
0
**
Notch3 +/+
Notch3 -/-

H

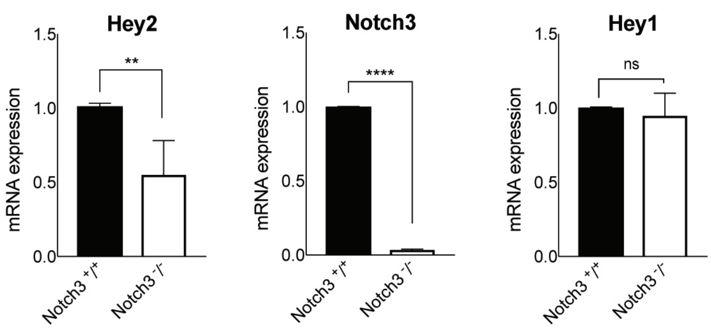
Hey2
Notch3
Hey1
mRNA expression
1.5
1.0
0.5
0.0
**
****
ns
Notch3 +/+
Notch3 -/-

Supplemental Figure 1:

Expression of the Atrial natriuretic peptide gene (NppA) (A) and the acyl-CoA dehydrogenase long and medium chain (Acadl, Acadm) as well as the proliferation-activated receptor-gamma (PPAR γ) (B) genes in n=2 control (Notch3+/+) and n=2 Notch3-/- hearts. Analysis of CD36 (C) and GLUT1 (D) distribution by immunohistochemistry in n=4 control and n=4 Notch3-/- heart sections and quantification (right panel). Size bar = 20μm. The mice used for these experiments were 10-14 months old. (E) Analysis of CD36 and GLUT1 distribution by immunohistochemistry in n=3 control and n=3 Notch3-/- heart sections and quantification (lower panel). One mouse of each genotype was analysed. The mice used for this experiment were two weeks old. Size bar = 50 μm (F) Analysis of CD36 and GLUT1 distribution by immunohistochemistry in n=3 control and n=3 Notch3-/- heart sections and quantification (lower panel). One mouse of each genotype was analysed. The mice used for this experiment were two months old. Size bar = 50 μm (G) Wheat germ agglutinate (WGA) (green) and DAPI (blue) staining of heart cross-sections from control (Notch3+/+) and Notch3-/- mice at two months of age. To the right, quantification of cardiomyocyte size from n=3 control and n=3 Notch3-/- heart sections. One mouse of each genotype was analysed. Size bar = 50 μm. (H) Expression of the Hey2, Notch3 and Hey1 genes in n=3 control and n=3 Notch3-/- hearts. The mice used for these experiments were 10-14 months old. *$p < 0.05$, **$p < 0.01$, ***$p<0.001$, ****$p < 0.0001$, ns = non-significant.

Supplemental Figure 2

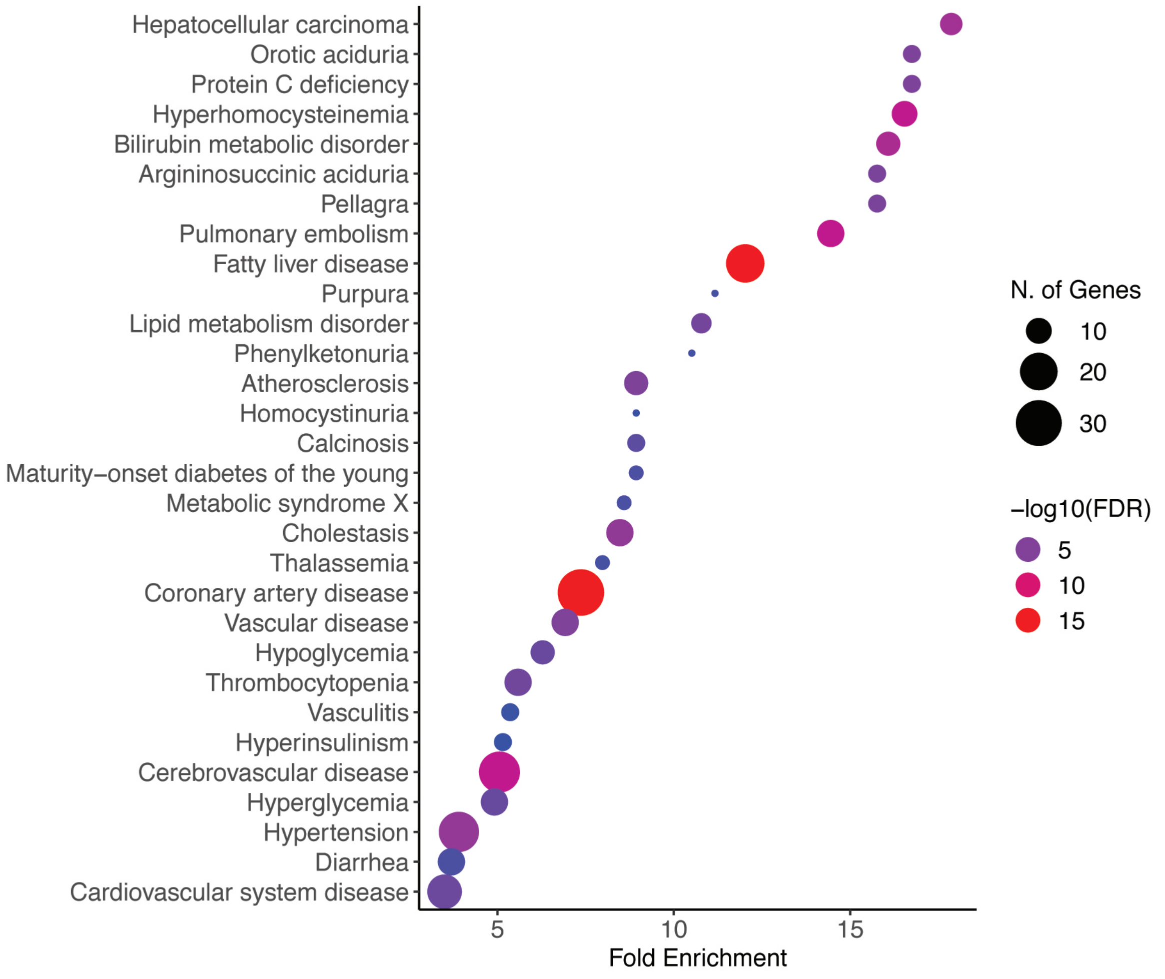

Supplemental Figure 2:
Barplot for Jensen disease pathway analysis, generated used the DEG from the comparison of Notch3-/- versus control (Notch3+/+) heart transcriptomes.

Supplemental Figure 3

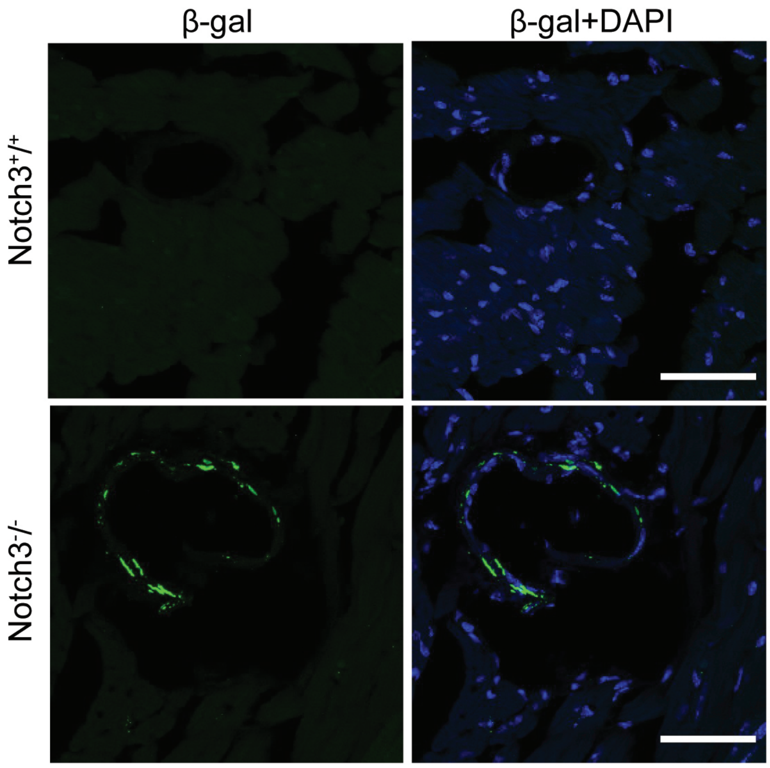

Supplemental Figure 3:
Expression of beta-galactosidase (β-gal; as a proxy for Notch3) in sections from Notch3-/- hearts (lower panel) but not from wildtype (upper panel) mice. The mice used for these experiments were 10-14 months old. Size bar = 50 μm.
